# Survival Outcomes According to Body Mass Index in Hepatocellular Carcinoma Patient: Analysis of Nationwide Cancer Registry Database

**DOI:** 10.1038/s41598-020-65460-9

**Published:** 2020-05-20

**Authors:** Boram Cha, Jung Hwan Yu, Young-Joo Jin, Young Ju Suh, Jin-Woo Lee

**Affiliations:** 1 Department of Internal Medicine, Inha University Hospital, Inha University School of Medicine, Incheon, South Korea; 2 Department of Biomedical Sciences, College of Medicine, Inha University, Incheon, South Korea

**Keywords:** Cancer, Gastroenterology, Risk factors

## Abstract

Background and Aims: Body mass index (BMI) is known to be closely related to the prognosis and mortality of various diseases. The aim of our study was to evaluate differences in post-treatment overall survival (OS) according to BMI with hepatocellular carcinoma (HCC) and to understand the meaning of BMI. Among the records of 10,578 HCC patients registered at the Korean Central Cancer Registry from 2008 through 2014, we selected Barcelona Clinic Liver Cancer (BCLC) 0, A, and B staged HCC patients (n = 4,926). HCC patients showed a good prognosis in the order of overweight, normal weight, obesity, and underweight. However, comparing normal-weight (BMI 18.5–24.9 kg/m^2^) to overweight (BMI 25–29.9 kg/m^2^) after propensity score matching (PSM), there was no significant difference in OS (*p* = 0.153). Overweight males had a better prognosis than normal-weight males (*p* = 0.014), but, normal-weight females had a better prognosis than overweight. To determine the gender-specific OS differences, we examined the differences according to the HCC treatment type. In males, overweight patients had better OS after transarterial chemoembolization (TACE) (*p* = 0.039) than normal-weight, but not after surgical resection (*p* = 0.618) nor radiofrequency ablation (*p* = 0.553). However, in females, all of those HCC treatments resulted in significantly better OS in normal-weight patients than overweight. In patients with HCC of BCLC stages 0–B, unlike females, overweight males had a better prognosis than normal-weight, especially among TACE-treated patients. Our results carefully suggest that the meaning of normal BMI in patients with HCC may have gender difference.

## Introduction

Hepatocellular carcinoma (HCC) is increasing in frequency and is a leading cause of cancer-related mortality worldwide^1^. Although tumor biology heterogeneity is an important determinant of prognosis in HCC patients, patient-level factors are also clearly important determinants. Therefore, established prognostic factors in HCC patients include not only tumor burden, but also the degree of liver dysfunction and the functional status of the patient. In practice, performance status indicators, such as the Eastern Cooperative Oncology Group (ECOG) score, are considered determinants of the treatment modality for HCC patients; however, ECOG score alone is not enough to assess the patient’s functional status, and further studies on other factors, such as body fat mass, are needed to reduce this limitation.

Body mass index (BMI), which is a value derived from the weight and height of an individual, has been shown to be highly correlated with body fat mass and is widely used to identify the subject’s physical condition. Furthermore, BMI is known to be associated with the prognosis of various diseases including malignant tumors^2^. In breast cancer, overweight and obese patients have a poorer prognosis than patients with normal body weight, because of increased estrogen hormone levels in adipocytes and the decreased effects of aromatase inhibitors in breast cancer patients with a higher BMI^3^. Meanwhile, it is known that the incidence of colorectal cancer is closely related to BMI, with overweight patients having the best prognosis among patients diagnosed with colorectal cancer^4^. Although there is no specific guideline for evaluating the HCC patient’s systemic condition, HCC patients with a low skeletal muscle component have a poor prognosis after surgical resection (SR)^5^, and patients who are notably lean may be cautious about the use of chemotherapy. Therefore, it is expected that the prognosis of HCC patients will be different according to their BMI. However, there is little research on how BMI relates to prognosis among HCC patients.

Therefore, we conducted a nationwide cancer registry-based cohort study to evaluate the differences in prognosis according to BMI, especially in normal and overweight HCC patients. Furthermore, we investigated differences in overall survival (OS) between normal and overweight BMI groups according to treatment modality and gender. Propensity score matching (PSM) was used to adjust for differences between the two groups.

## Materials and Methods

### Database extraction

A nationwide cancer registry, the Korea Central Cancer Registry (KCCR), was initiated by the Ministry of Health and Welfare, South Korea in 1980. HCC patients were extracted from the KCCR registry using code C22.0 of the International Classification of Disease 10^th^ edition (ICD-10) coding system. The National Cancer Center and Korean Liver Cancer Study group have systemically organized the KCCR database annually by applying a random sample audit method. During the 2008–2014 periods, every year between 11,547 and 12,194 patients have been registered at 47 to 54 hospitals. Clinical data for 83,231 patients were examined. Of these, 10,811 (13%) patient records, which included an additional 3% considering the presence of sampling errors, were randomly abstracted. Finally, clinical data for 10,578 HCC patients were initially included in this study.

Mortality data for the enrolled patients were obtained from the Korean National Statistics Office. Initial treatment dates were determined based on KCCR records. For survival analysis, follow-up durations were calculated from the date of initial treatment to the date of death or to December 31, 2016. The study was approved by the Institutional Review Board of Inha University Hospital, Incheon, South Korea (Approval number: INHAUH 2018-09-003-001).

### Study subjects

**Figure 1:**
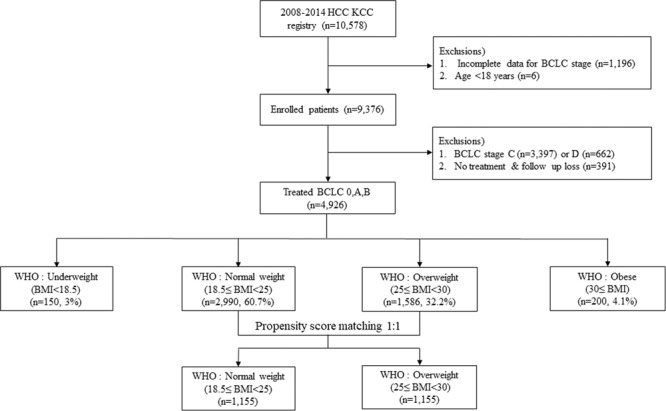
Flowsheet of the enrolled all patients (n = 10,578).

A schematic flowsheet for the inclusion and categorization of the study subjects is shown in Fig. 1. Of the 10,578 patients in KCCR, patients with incomplete data for Barcelona Clinic Liver Cancer (BCLC) stage (n = 1,196) and with an age <18 years (n = 6) were excluded. Of the remaining 9,376 patients, those with BCLC stage C (n = 3,397) or D (n = 662), and those who underwent liver transplantation (n = 52) or other treatments (chemotherapy, radiotherapy, or Sorafenib) (n = 72) were also excluded. Moreover, patients with no available data for treatment type or without follow-up data (n = 391) were excluded. Finally, BCLC 0, A, or B staged HCC patients (n = 4,926) treated with SR, radiofrequency ablation (RFA), or transarterial chemoembolization (TACE) were analyzed in this retrospective cohort study. Among those 4,926 HCC patients, 150 (3.0%), 2,990 (60.7%), 1,586 (32.3%), and 200 (4.1%) patients had BMI values corresponding to underweight, normal weight, overweight, and obese categories, respectively.

By using the KCCR database, we obtained data for the following variables: age, gender, weight and height, smoking history, comorbidities of hypertension (HTN) or diabetes mellitus (DM), HCC etiology, alanine aminotransferase (ALT), albumin, total bilirubin, prothrombin time (PT), serum sodium (Na), serum creatinine (Cr), total cholesterol, alpha-fetoprotein (AFP), Child-Turcotte-Pugh (CTP) class, model for end-stage liver disease (MELD) scores, MELD-sodium (MELD-Na) scores, tumor number and size, BCLC stage, and treatment type.

### Statistical analyses

The clinical characteristics of HCC study subjects were expressed as medians (ranges) for continuous variables, and numbers (percentages) for categorical variables. Differences between categorical or continuous variables were analyzed using the Student’s *t* test, the *chi-*squared test, or Fisher’s exact test. To compare each characteristics on Table 1, we used Kruskal wallis H test.

**Table 1:** Baseline clinical characteristics of study subjects according to WHO criteria.

Variables (n = 4,926)	Under-weight (n = 150, 3%)	Normal Weight (n = 2,990, 60.7%)	Overweight (n = 1,586, 32.2%)	Obese (n = 200, 4.1%)	p*
Age (year) ^§^	63 (27–87)	60 (24–91)	60 (18–91)	60 (35–82)	7.7e-5
Gender (male), n (%)	121 (80.1)	2,332 (78)	1,220 (76.9)	121 (60.5)	1.4e-9
BMI (kg/m^2^) ^§^	17.5 (11.8–18.5)	22.4 (18.5–25.0)	26.9(25.0–30.0)	31.9(30.0–40.6)	1.8e-10
Smoking (pack/year)	25 (5–70)	21 (0.1–200)	22.5 (0.6–200)	20 (0.5–129)	0.403
HTN, n (%)	28 (18.5)	939 (31.4)	638 (40.2)	105 (52.7)	1.5e-8
DM, n (%)	33 (21.9)	730 (24.4)	408 (25.7)	61 (30.7)	0.140
Cause of HCC, n (%)					
HBV	83 (55)	1842 (61.6)	1,007 (63.5)	117 (58.5)	0.041
HCV	26 (17.2)	395 (13.2)	203 (12.8)	24 (12.2)	0.592
NBNC	2 (1.3)	81 (2.7)	59 (3.7)	11 (5.4)	0.108
Alcohol	50 (33.1)	882 (29.3)	449 (28.3)	47 (23.4)	0.174
ALT (U/L)	64.8(4.7)	58.4(1.5)	57.9(1.9)	54.8(4.2)	0.945
Albumin (g/dL)^§^	3.7 (1.0–5.2)	3.9 (0.5–5.6)	3.9(0.8–5.5)	3.8(2.1–5.0)	0.001
Bilirubin (mg/dL)^§^	1.1 (0.2–16.3)	1.1 (0.1–31.3)	1.1(0.2–30.8)	1.1(0.3–5.5)	0.031
PT, INR^§^	1.1 (0.8–2.0)	1.1 (0.1–71.7)	1.1(0.8–1.9)	1.1(0.9–1.7)	0.018
Cr (mg/dL)^§^	0.9 (0.3–3.8)	1.0 (0.2–16.1)	1.0(0.3–13.5)	1.0(0.4–9.3)	0.001
Na (mEq/L)^§^	137.8 (126–144)	139.3 (100–157)	139.7(103–160)	140.0(130–149)	1.8E-7
T.chol (mg/dL)^§^	147.0 (67–238)	157.1 (1.2–542)	161.8(15–462)	165.3(0.74–459)	0.010
AFP (ng/dL)^§^	1616 (1.1–60500)	2267 (0.4–481276)	1571(0.7–435220)	998(1.3–46213)	0.075
CTP class, n (%)					0.063
A	119 (78.8)	2559 (85.6)	1,375 (86.7)	173 (86.3)	
B	32 (21.2)	428 (14.3)	208 (13.1)	26 (13.2)	
MELD score^§^	8.7 (6–21)	8.9 (6–58)	8.8(6–45)	9.2(6–22)	0.067
MELD-Na^§^	10.8 (6–25)	10.1 (6–56)	9.8(6–45)	10.1(6–26)	0.001
Tm, n (%)					0.366
Solitary	100 (66.2)	2,135 (71.4)	1,124 (70.9)	140 (69.8)	
Multiple	50 (23.8)	855 (28.6)	461 (29.1)	40 (20.2)	
Tm size (cm)	4.2(0.6–15)	3.7(0.1–23)	3.4(0.2–22)	3.4(0.5–19)	0.164
BCLC, n (%)					0.033
0	16 (10.6)	350 (11.7)	208 (13.1)	20 (9.8)	
A	91 (60.3)	2,060 (68.9)	1,080 (68.1)	139 (69.3)	
B	44 (29.1)	580 (19.4)	298 (18.8)	42 (21)	
Treatment, n (%)					0.001
SR	37 (24.5)	858 (28.7)	430 (27.1)	41 (20.5)	
RFA	14 (9.3)	505 (16.9)	287 (18.1)	35 (17.6)	
TACE	69 (45.7)	1,363 (45.6)	755 (47.6)	103 (51.7)	
Follow up duration month) ^§^	35.4 (0.5–103.7)	46.9 (0.0–107.8)	48.1(0.1–107.5)	45.2(1.4–106.7)	2.5e-7
Ascites (grade)					0.001
0	122 (80.8)	2,649 (88.6)	1,434 (90.4)	186 (93.2)	
1	23 (15.2)	260 (8.7)	119 (7.5)	10 (4.9)	
2	6 (4)	81 (2.7)	33 (2.1)	4 (2)	

^§^Median (range).

^*^p values were calculated using the Kruskal-Wallis H test.

WHO, World Health Organization; BMI, body mass index; HTN, hypertension; DM, diabetes mellitus; HCC, hepatocellular carcinoma; HBV, hepatitis B virus; HCV, hepatitis C virus; NBNC, non-hepatitis B and non-hepatitis C; ALT, aminotransferase; PT, prothrombin time; INR, international normalized ratio; Cr, creatinine; Na, sodium; T.chol, total cholesterol; AFP, alpha-fetoprotein; CTP, Child-Turcotte-Pugh; MELD, model for end-stage liver disease; Tm, tumor; BCLC, Barcelona Clinic Liver Cancer; SR, surgical resection; RFA, radiofrequency ablation; TACE, transarterial chemoembolization.

To investigate the association between treatment selection and clinical outcomes in an observational and nonrandomized study, we performed PSM analysis to reduce the imbalance in the distribution of the demographic and clinical characteristics between the two groups of normal weight (18.5 ≤ BMI < 25) and overweight (25 ≤ BMI < 30) patients. Propensity scores for the two groups were estimated of the demographic and clinical variables such as pretreatment characteristics, including sex, BMI, smoking, HTN, DM, cause of underlying liver disease (chronic hepatitis B, chronic hepatitis C, alcohol, and unknown), serum albumin, serum total bilirubin, PT (INR), serum creatinine, serum sodium, AFP level, CTP class, MELD score, tumor number, tumor size, and BCLC stage (Table 2). Furthermore, general characteristics in normal weight and overweight patients after PSM divided in females and males was also compared (Supplementary table 2). The PSM was implemented using the 1:1 nearest algorithm with a caliper width of 0.03 multiplied by the standard deviation of the value. The PSM analysis was performed using R software v. 3.5.0 (https://www.r-project.org/, ‘MatchIt’ package).

**Table 2:** General characteristics after PSM.

	Normal weight (n = 1,155)	Overweight (n = 1,155)	p*
Age, mean(SD)	59.63(10.59)	59.7(10.32)	0.870
Gender, n(%)			0.881
Female	256	260	
Male	899	895	
Smoke, n(%)			0.900
No	655	659	
Yes	500	496	
HTN, n(%)			0.521
No	703	719	
Yes	452	436	
DM, n(%)			0.885
No	866	870	
Yes	289	285	
HCC cause, n(%)			0.996
HBV	733	737	
HCV	130	132	
NBNC	20	22	
Alcohol	125	118	
Mixed (HBV + HCV)	10	11	
Unknown	137	135	
Laboratory findings ALT, mean(SD)	45.6(1.95)	49.38(2.08)	0.644
Albumin, mean(SD)	3.96(0.57)	3.95(0.56)	0.659
Platelet, mean(SD)	142.61(68.01)	141.39(69.14)	0.667
PT(INR), mean(SD)	1.11(0.14)	1.11(0.15)	0.318
Cr, mean(SD)	0.93(0.59)	0.92(0.42)	0.652
Na, mean(SD)	139.72(3.62)	139.73(3.07)	0.890
Total cholesterol, mean(SD	160.22(37.98)	160.73(37.02)	0.745
AFP, mean(SD)	1162.94(5992.19)	1132.1(7435.5)	0.913
CTP class, n(%)			0.639
1	1034	1026	
2	121	129	
MELD score, mean(SD)	8.61(2.51)	8.65(2.5)	0.648
MELD-Na, mean(SD)	9.54(3.16)	9.6(3.16)	0.626
Tumor number, n(%)			0.918
Single	812	814	
Multiple	343	341	
Tumor size, mean(SD)	288.8 (43.0)	288.8 (37.3)	0.911
BCLC stage, n(%)			0.968
0	163	160	
A	776	775	
B	216	220	

*p values were calculated using the *t*-test or Fisher’s exact test.

PSM, propensity score matching; SD, standard deviation; HTN, hypertension; DM, diabetes mellitus; HCC, hepatocellular carcinoma; HBV, hepatitis B virus; HCV, hepatitis C virus; NBNC, non-hepatitis B and non-hepatitis C; ALT, aminotransferase; PT, prothrombin time; INR, international normalized ratio; Cr, creatinine; Na, sodium; AFP, alpha-fetoprotein; CTP, Child-Turcotte-Pugh; MELD, model for end-stage liver disease; BCLC, Barcelona Clinic Liver Cancer.

The OS rates were estimated using the Kaplan-Meier method. The difference between the OS curves of groups was tested using the log-rank test. Two-tailed *p*-values of <0.05 were considered statistically significant, and the statistical analysis was performed using SPSS v19.0 (SPSS Inc, Chicago, IL, USA).

## Results

### Baseline characteristics

The baseline clinical characteristics of study subjects according to the World Health Organization (WHO) criteria and BMI category are presented in Table 1. Based on BMI, the median age was 63 years (range, 27–87 years), 60 years (range, 24–91 years), 60 years (range, 18–91 years), and 60 years (range, 35–82 years) in the underweight, normal weight, overweight, and obese groups, respectively. The proportion that was male was 121 (80.1%), 2,332 (78%), 1,220 (76.9%), and 121 (60.5%) in each respective group, and there was a significantly low percentage of males in the obese group. The median BMI in each of the respective groups was 17.5 (range, 11.8–18.5 kg/m^2^), 22.4 (range, 18.5–25 kg/m^2^), 26.9 (range, 25–30 kg/m^2^), and 31.9 (range, 30–40.6 kg/m^2^). The proportion of accompanying HTN was highest in obese group (52.7%); however, the incidence of DM was not significantly different among the four groups (*p* = 0.14). Remnant liver function by CTP class did not significantly differ among the four groups (*p* = 0.063).

In terms of HCC etiology, the frequency of hepatitis B virus (HBV) infection was comparatively high in the normal (61.6%) and overweight (63.5%) groups; in contrast, hepatitis C virus (HCV) (*p* = 0.592), non-B non-C (NBNC) hepatitis (*p* = 0.108), and alcohol (*p* = 0.174) frequencies were not significantly different among the groups. With regard to tumor features, there was no statistically significant difference in the proportion of solitary HCC (*p* = 0.366) or in tumor size (*p* = 0.164). The proportion of patients treated with SR was significantly low in the obese group and RFA was significantly low in the underweight group. However, BCLC stage showed no significant differences among the groups (*p* = 0.33). We evaluated the proportion of ascites grades to assess whether the existence of ascites can affect BMI; the proportion of the lower ascites grade was significantly high in the obese group.

### Overall survival rate of HCC patients according to BMI before and after PSM

We selected WHO criteria rather than Asian criteria, because, according to Asian criteria, OS between normal weight (18.5 ≤ BMI < 23) and overweight (23 ≤ BMI < 25) groups was not different and because this is the same BMI range as that for normal weight (18.5 ≤ BMI < 25) individuals in the WHO criteria (Supplementary Fig. 2).

**Figure 2:**
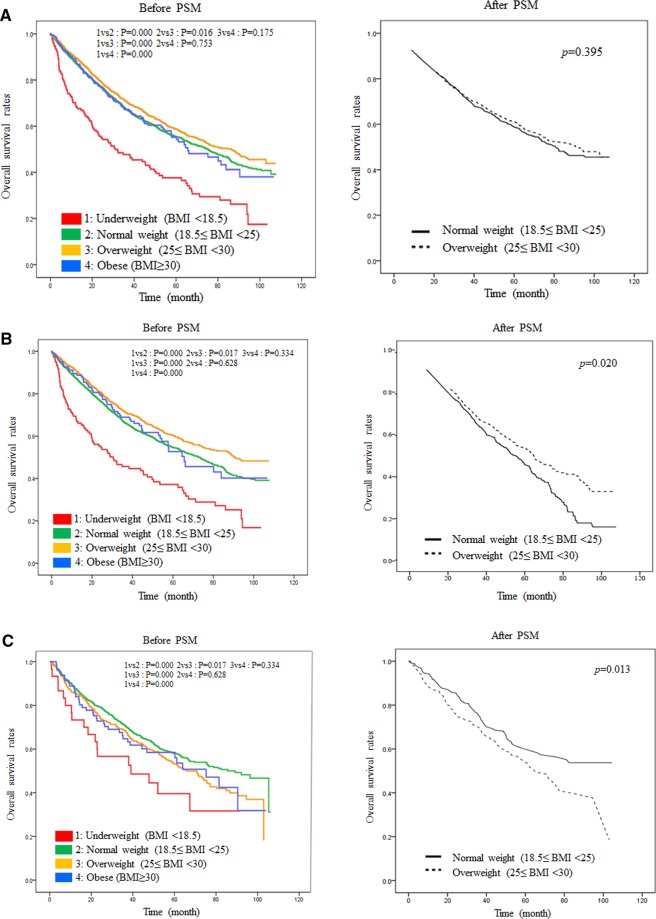
Overall survival rate compared to before and after PSM. (**A**) overall survival rate without gender difference (**B**) overall survival rate in males (**C**) overall survival rate in females; comparison between normal weight (18.5 ≤ BMI < 25) vs overweight (25 ≤ BMI < 30) after PSM; PSM, propensity score matching.

After performing PSM, 1,155 HCC patients with BCLC stages 0-B were allocated to each of the normal weight and overweight groups. Before PSM, the underweight group showed the lowest OS among the four weight groups, whereas the overweight group had a significantly high OS compared to that of the normal group (overweight *vs*. normal, *p* = 0.016). However, this significant difference disappeared after PSM (*p* = 0.395) (Fig. 2A). To investigate why the overweight group would exhibit a better survival rate, we analyzed the data by gender. Among the male patients, the overweight group had a significantly higher OS before PSM, and the OS of the overweight group was still significantly higher after PSM (Fig. 2B). However, among the female patients, the normal weight group had a significantly higher OS than the overweight group both before and after PSM (Fig. 2C).

### Overall survival rate of HCC patients according to treatment types after PSM

**Figure 3:**
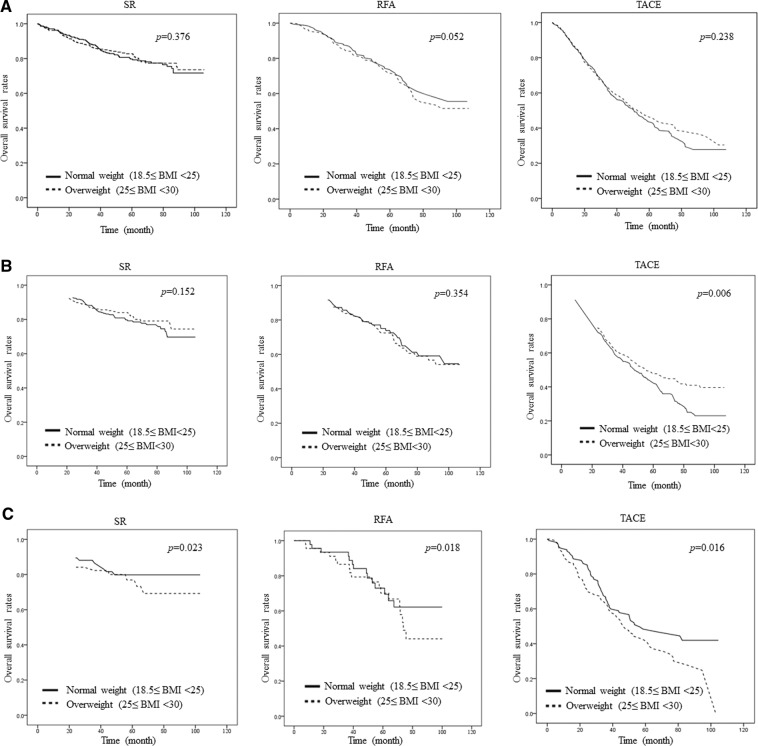
Differences in overall survival rate according to treatment type after PSM. (**A**) overall survival rate without gender difference (**B**) overall survival rate in males (**C**) overall survival rate in females; comparison between normal weight (18.5 ≤ BMI < 25) vs overweight (25 ≤ BMI < 30) after PSM; PSM, propensity score matching; SR, surgical resection; RFA, radiofrequency ablation; TACE, trans-arterial chemoembolization.

As shown in Fig. 2, there was a gender-based difference according to BMI group in HCC patients. To investigate other factors that could affect the gender difference according to BMI, we separately analyzed by HCC treatment type (SR, RFA, and TACE). When the combined gender group of patients were analyzed according to treatment type, there were no significant differences between normal weight and overweight patients in the SR (*p* = 0.376), RFA (*p* = 0.052), and TACE (*p* = 0.238) treatment groups (Fig. 3A). However, among the male HCC patients, the TACE-treated group showed significantly higher OS than that of the overweight group (*p* = 0.006) (Fig. 3B). Among the female patients, all three HCC treatment types had significantly higher OS in the normal weight groups than in the respective overweight groups (SR; *p* = 0.023, RFA; *p* = 0.018, TACE; *p* = 0.016) (Fig. 3C).

## Discussion

In our study, based on data from the KCCR database, patients with HCC showed different OS rates according to their BMI, and those that were categorized as overweight had the best prognosis. However, there was no significant difference in OS rates when overweight and normal-weight patients were compared after PSM. When we analyzed patient prognosis according to gender and treatment method, overweight males had a better prognosis than that of normal-weight patients, especially among TACE-treated patients. On the other hand, normal-weight female patients had a better prognosis than that of overweight patients, regardless of which treatment (SR, RFA, and TACE) they received. The results of this study are meaningful because the prognosis according to BMI findings were derived from a comprehensive analysis that included using PSM on a large-scale dataset obtained from a random sample audit of a nationwide database; in addition, subgroup analyses based on gender and treatment method were performed.

The definitions of obesity and overweight according to BMI are somewhat different according to the region and organization being examined. Currently, the WHO and the Asian BMI criteria are the most commonly used. In the Asian criteria, the agreed cut-off for inclusion in the overweight category is 23.0 kg/m^2^ ^6^. Whereas, the WHO criteria cut-off for the overweight category is 25 kg/m^2^, which means the WHO normal weight category includes both normal weight and overweight categories of the Asian criteria (Supplementary table 1). Although there are pros and cons associated with each of these criteria sources, we used the WHO standard as it is more widely used; nonetheless, the results analyzed according to the Asian criteria are presented in Supplementary fugure 1 and 2.

Obesity and energy imbalance components are established risk factors for the incidence of several cancers, including HCC^7,8^. However, the role of obesity in the survival outcome of cancer patients is unclear. As a negative aspect of obesity (BMI ≥ 30 kg/m^2^) in cancer, a meta-analysis report showed that a higher BMI decreased survival among prostate, breast, and colorectal cancer patients^2–9^. Nonetheless, most of those studies were not specifically designed to evaluate the relationship between BMI and survival. As a positive aspect of obesity, among colorectal cancer patients, an overweight group (25 kg/m^2^ ≤ BMI < 30 kg/m^2^) showed a significantly lower mortality risk than patients who were categorized as ‘low-normal’ weight (18.5 kg/m^2^ ≤ BMI < 23 kg/m^2^) both in all-cause mortality and in colorectal cancer-specific mortality^4^. Also among 4,010 distant metastasis patients, both overweight (HR = 0.84; *p* < 0.001) and obese patients (HR = 0.676; 95% *p* < 0.001) exhibited reduced risks of all-cause mortality in a multivariable analysis^10^. Our study results are consistent with some of the positive aspects of overweight status increasing OS in HCC patients. However, when comparing the survival rate associated with BMI in cancer patients, patients with a low BMI are likely to have a poor prognosis due to the impact of cachexia. Considering this, future studies are needed to investigate the incidence of cachexia in HCC patients whose prediagnosis BMI is identified, and to examine the impact of cachexia on survival rate.

In our quest to determine the reason for the gender difference exhibited in our study, we wondered whether the definition of BMI is appropriate. BMI is undoubtedly the most frequently used proxy of adiposity/obesity in large epidemiological studies in both healthy and diseased populations. Despite its wide use, which reflects its level of convenience since it only requires the measurement of height and mass, BMI has been frequently criticized as having various deficiencies when used as a measure of obesity^11^ both in healthy and diseased populations^12^. The difference between male and female HCC patients observed in our study may be partially explained by the sex-related difference in the relationships of BMI to total body fat; specifically, even with the same BMI, males are known to have a higher muscle percentage than females^13,14^. Thus, with regard to the limitations of BMI interpretation, the meaning of ‘overweight’ in a male may reflect not just a weight increase, but specifically, an increase in muscle weight. A comparatively high muscle composition might contribute to an increased power to resist HCC.

One of the other issues with BMI is that it does not reflect some changes, particularly that in the presence of sarcopenia, which is characterized by reduced muscle mass and increased adiposity. Recently, sarcopenia has garnered attention as a new and promising prognostic factor in various malignancies, including HCC^15,16^. Sarcopenia presence is also related to poor survival in patients with liver cirrhosis^17,18^. Unfortunately, we did not properly assess patient muscle mass in this study. However, based on previous studies on sarcopenia, the presence of sarcopenia is considered to have a significant effect on the prognosis of HCC patients, even in patients with the same BMI^19^. In this study, we cautiously predict that there were fewer patients with sarcopenia in the overweight group than in the normal-weight group, especially among male patients.

Some limitations of this study should be taken into account. First, we could not completely eliminate the inherent selection bias due to a retrospective study design. However, we tried our best to minimize the potential confounding factors by using a random sampling audit method in a large-scale nationwide cancer registry, as well we applied PSM. Second, BMI was measured at the time of diagnosis by as a nationwide cancer registration-based cohort data, and there may be an effect of cachexia by HCC on BMI measurement, but there is a disadvantage in that this effect cannot be accurately evaluated. Third, due to the insufficient data related to patient comorbidities in the KCCR database, we could not evaluate the effect of all comorbidities on the survival rate of HCC patients. However, patients’ DM and HTN medical history and smoking history could be recruited, and our analysis revealed that these factors were not prognostic of OS in the enrolled patients. Fourth, we could not separately analyze the body composition of patients because all of the raw data were derived from the KCCR database. This is important because, based on the assumption that, in same BMI group, males have higher muscle composition and lower body fat than females. To examine this limitation, a hospital cohort of patients with specific analysis of the effect of body composition (visceral fat and muscle) on HCC survival is warranted.

In conclusion, the prognosis for overweight HCC patients was the best among the HCC patients in the nationwide cancer registration-based cohort data. However, the OS rates of normal-weight and overweight patients after PSM were not significantly different. Subgroup analysis revealed that male patients had a better prognosis when overweight, especially among TACE-treated patients. On the other hand, normal-weight female patients had a better prognosis than that of overweight patients. Although the difference between male and female patients is thought to be due to sex-based differences in body mass composition, follow-up studies are needed.

## Supplementary information

Supplementary Information.
