# Supplementary material for: Survival Outcomes According to Body Mass Index in Hepatocellular Carcinoma Patient: Analysis of Nationwide Cancer Registry Database
Source: Sci Rep. 2020 May 20;10:8347. doi: 10.1038/s41598-020-65460-9 (PMC7239934; doi:10.1038/s41598-020-65460-9)
Supplement: Supplementary file 1 — Supplementary Information. [file 41598_2020_65460_MOESM1_ESM.docx]

**Title:**

**Survival Outcomes According to Body Mass Index in Hepatocellular Carcinoma Patient: Analysis of Nationwide Cancer Registry Database**

**Authors**

Boram Cha^1^, Jung Hwan Yu^1^*****, Young-Joo Jin^1^, Young Ju Suh^2^, Jin-Woo Lee^1^*****

**Affiliations of authors:**

1. Department of Internal Medicine, Inha University Hospital, Inha University School of Medicine, Incheon, South Korea

2. Department of Biomedical Sciences, College of Medicine, Inha University, Incheon, South Korea

*** Corresponding author**:

**
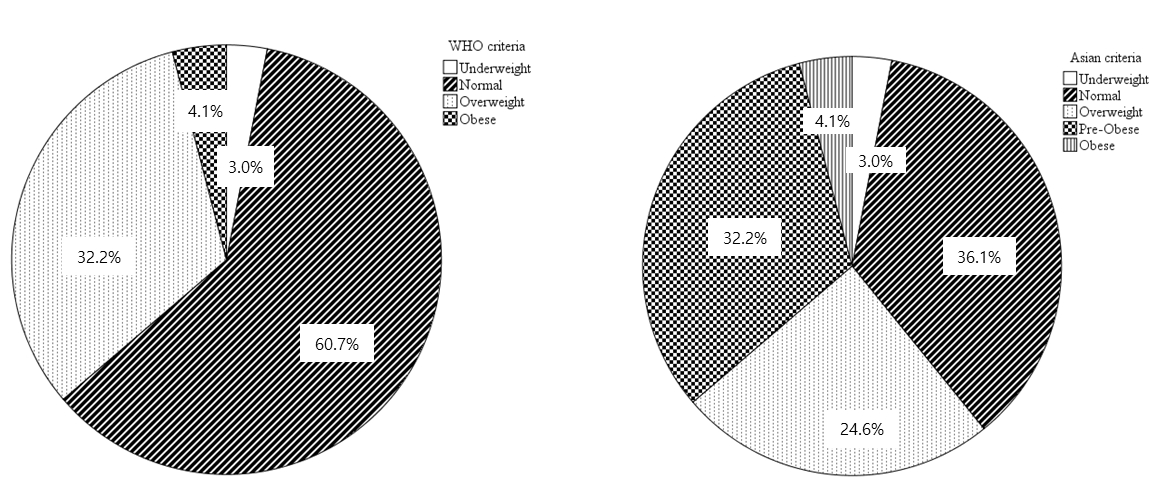
Supplementary Figures 1**

**Supplementary figure 1. Percentages of patients according to WHO criteria and Asian criteria.** WHO criteria: Underweight (BMI<18.5), Normal weight (18.5≤BMI<25), Overweight (25≤BMI<30), Obese (30≤ BMI); Asian criteria: Underweight (BMI<18.5), Normal weight (18.5≤ BMI<23), Overweight (23≤BMI<25), Pre-Obese (25≤BMI<30), Obese (30≤BMI)

**Supplementary Figures 2**


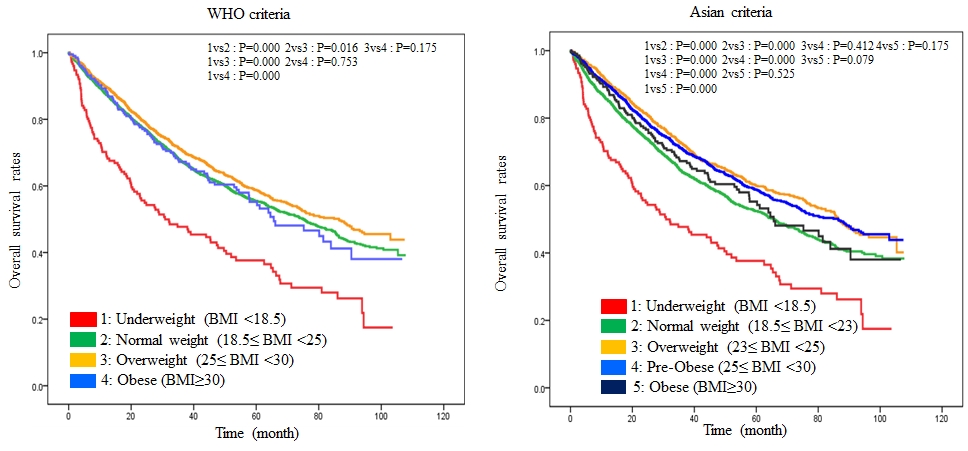


**Supplementary figure 2. Differences in overall survival rate between WHO and Asian criteria.** WHO criteria: Underweight (BMI<18.5), Normal weight (18.5≤BMI<25), Overweight (25≤BMI<30), Obese (30≤BMI); Asian criteria: Underweight (BMI<18.5), Normal weight (18.5≤ BMI<23), Overweight (23≤ BMI<25), Pre-Obese (25≤BMI<30), Obese (30≤BMI)

**Supplementary Table**

| **Supplementary Table 1. Difference of WHO and Asian criteria on BMI cut-off category**   \| **Nutritional Status** \| **WHO criteria (kg/m^2^)** \| **Asian criteria (kg/m^2^)** \| \| --- \| --- \| --- \| \| Underweight \| < 18.5 \| < 18.5 \| \| Normal \| 18.5-24.9 \| 18.5-22.9 \| \| Overweight \| 25-29.9 \| 23-24.9 \| \| Pre-Obese \|  \| 25-29.9 \| \| Obese \| ≥ 30 \| ≥ 30 \|   WHO, World Health Organization; BMI, body mass index  **Supplement Table 2. General characteristics in normal weight and overweight patients after PSM divided in females and males**   \|  \| **Normal weight**  **(n=1,155)** \| \| \| \| \|  \| \| **Overweight**  **(n=1,155)** \| \|  \| \| --- \| --- \| --- \| --- \| --- \| --- \| --- \| --- \| --- \| --- \| --- \| \|  \| **Male**  **(n=898)** \| \| **Female**  **(n=257)** \| \| \| ***p**** \| \| **Male**  **(n=895)** \| **Female**  **(n=260)** \| ***p**** \| \| Age, mean(SD) \| 59.52(10.2) \| \| 59.95(11.9) \| \| \| 0.562 \| \| 57.92(9.8) \| 65.8(9.6) \| 0.52 \| \| Smoke pack year,  mean(SD) \| 27.9(19.6) \| \| 12.6(10.8) \| \| \| <0.01 \| \| 27.37(21.9) \| 28.7(33.2) \| 0.223 \| \| HTN, n(%) \| \| 357 \| \| 94 \| 0.39 \| \| 316 \| \| 120 \| 0.02 \| \| DM, n(%) \| \| 238 \| \| 51 \| 0.03 \| \| 219 \| \| 66 \| 0.81 \| \| HCC cause, n(%) \| \|  \| \|  \|  \| \|  \| \|  \|  \| \| HBV \| \| 566 \| \| 185 \| 0.03 \| \| 607 \| \| 152 \| 0.02 \| \| HCV \| \| 123 \| \| 47 \| 0.10 \| \| 102 \| \| 67 \| <0.01 \| \| NBNC \| \| 42 \| \| 10 \| 0.57 \| \| 54 \| \| 14 \| 0.59 \| \| Alcohol \| \| 295 \| \| 13 \| <0.01 \| \| 324 \| \| 7 \| <0.01 \| \| ALT, means(SD) \| \| 46.3(2.3) \| \| 43.1(3.5) \| \| 0.96 \| \| 51.0(2.4) \| 43.7(3.9) \| 0.40 \| \| Albumin, means(SD) \| \| 3.9(0.6) \| \| 3.9(0.6) \| \| 0.64 \| \| 4.0(0.6) \| 3.8(0.5) \| 0.99 \| \| Platelet, means(SD) \| \| 144k(68k) \| \| 135k(66k) \| \| 0.05 \| \| 145k(70k) \| 128k(64k) \| 0.09 \| \| PT(INR), means(SD) \| \| 1.11(0.1) \| \| 1.12(0.1) \| \| 0.21 \| \| 1.1(0.1) \| 1.2(0.1) \| 0.25 \| \| Cr, means(SD) \| \| 0.98(0.6) \| \| 0.77(0.5) \| \| <0.01 \| \| 0.97(0.1) \| 0.76(0.2) \| 0.29 \| \| Na, means(SD) \| \| 139(3.4) \| \| 140(4.1) \| \| 0.17 \| \| 139.7(2.8) \| 139.9(3.8) \| 0.04 \| \| Total cholesterol, means(SD) \| \| 158(36.9) \| \| 166(40.8) \| \| <0.01 \| \| 162(37) \| 155(35) \| 0.78 \| \| AFP, means(SD) \| \| 970(190) \| \| 1844(429) \| \| 0.04 \| \| 1052(751) \| 1408(716) \| 0.33 \| \| CTP class, n(%)  1  2 \| \| 801  97 \| \| 232  24 \| \| 0.51 \| \| 801  94 \| 225  35 \| 0.181 \| \| MELD score, means(SD) \| \| 9(2.6) \| \| 8(2.2) \| \| 0.03 \| \| 9(2.5) \| 9(2.4) \| 0.46 \| \| MELD-Na, means(SD) \| \| 10(3.2) \| \| 9(2.9) \| \| <0.01 \| \| 10(3.2) \| 10(3.2) \| 0.483 \| \| Tumor number, n(%) \| \|  \| \|  \| \| 0.03 \| \|  \|  \| 0.03 \| \| Single \| \| 612 \| \| 199 \| \|  \| \| 614 \| 200 \|  \| \| Multiple \| \| 286 \| \| 57 \| \|  \| \| 281 \| 60 \|  \| \| Tumor size(cm), mean(SD) \| \| 3.6(2.8) \| \| 3.3(2.9) \| \| 0.10 \| \| 3.6(2.7) \| 3.1(2.1) \| 0.05 \| \| BCLC stage, n(%) \| \|  \| \|  \| \| 0.04 \| \|  \|  \| 0.01 \| \| 0 \| \| 117 \| \| 46 \| \|  \| \| 115 \| 45 \|  \| \| A \| \| 602 \| \| 173 \| \|  \| \| 595 \| 180 \|  \| \| B \| \| 179 \| \| 37 \| \|  \| \| 185 \| 35 \|  \|   PSM, propensity score matching; SD, standard deviation; HTN, hypertension; DM, diabetes mellitus; HCC, hepatocellular carcinoma; HBV, hepatitis B virus; HCV, hepatitis C virus; NBNC, non-hepatitis B and non-hepatitis C; ALT, aminotransferase; PT, prothrombin time; INR, international normalized ratio; Cr, creatinine; Na, sodium; AFP, alpha-fetoprotein; CTP, Child-Turcotte-Pugh; MELD, model for end-stage liver disease; BCLC, Barcelona Clinic Liver Cancer |
| --- | --- | --- | --- | --- | --- | --- | --- | --- | --- | --- | --- | --- | --- | --- | --- | --- | --- | --- | --- | --- | --- | --- | --- | --- | --- | --- | --- | --- | --- | --- | --- | --- | --- | --- | --- | --- | --- | --- | --- | --- | --- | --- | --- | --- | --- | --- | --- | --- | --- | --- | --- | --- | --- | --- | --- | --- | --- | --- | --- | --- | --- | --- | --- | --- | --- | --- | --- | --- | --- | --- | --- | --- | --- | --- | --- | --- | --- | --- | --- | --- | --- | --- | --- | --- | --- | --- | --- | --- | --- | --- | --- | --- | --- | --- | --- | --- | --- | --- | --- | --- | --- | --- | --- | --- | --- | --- | --- | --- | --- | --- | --- | --- | --- | --- | --- | --- | --- | --- | --- | --- | --- | --- | --- | --- | --- | --- | --- | --- | --- | --- | --- | --- | --- | --- | --- | --- | --- | --- | --- | --- | --- | --- | --- | --- | --- | --- | --- | --- | --- | --- | --- | --- | --- | --- | --- | --- | --- | --- | --- | --- | --- | --- | --- | --- | --- | --- | --- | --- | --- | --- | --- | --- | --- | --- | --- | --- | --- | --- | --- | --- | --- | --- | --- | --- | --- | --- | --- | --- | --- | --- | --- | --- | --- | --- | --- | --- | --- | --- | --- | --- | --- | --- | --- | --- | --- | --- | --- | --- | --- | --- | --- | --- | --- | --- | --- | --- | --- | --- | --- | --- | --- | --- | --- | --- | --- | --- | --- | --- | --- | --- | --- | --- | --- | --- | --- | --- | --- | --- | --- | --- | --- | --- | --- | --- | --- | --- | --- | --- | --- | --- | --- | --- | --- | --- | --- | --- | --- | --- | --- | --- | --- | --- | --- | --- | --- | --- | --- | --- | --- | --- | --- | --- | --- | --- | --- | --- | --- | --- | --- | --- | --- | --- | --- | --- | --- | --- | --- | --- | --- | --- | --- | --- | --- | --- | --- | --- | --- | --- | --- | --- | --- | --- | --- | --- | --- | --- | --- | --- | --- | --- | --- | --- | --- | --- | --- | --- | --- | --- | --- | --- | --- | --- | --- | --- | --- | --- | --- | --- | --- | --- | --- | --- | --- | --- | --- | --- | --- | --- | --- | --- | --- | --- | --- | --- | --- | --- | --- | --- |
